# Graphene/TiO_2_ Heterostructure Integrated with a Micro-Lightplate for Low-Power NO_2_ Gas Detection

**DOI:** 10.3390/s25020382

**Published:** 2025-01-10

**Authors:** Paniz Vafaei, Margus Kodu, Harry Alles, Valter Kiisk, Olga Casals, Joan Daniel Prades, Raivo Jaaniso

**Affiliations:** 1 Institute of Physics, University of Tartu, EE-50411 Tartu, Estonia; paniz.vafaei@ut.ee (P.V.); margus.kodu@ut.ee (M.K.); harry.alles@ut.ee (H.A.); valter.kiisk@ut.ee (V.K.); 2 MIND-IN2 UB, Department of Electronics and Biomedical Engineering, Universitat de Barcelona, E-08028 Barcelona, Spain; olga.casals@ub.edu; 3 Laboratory for Emerging Nanometrology (LENA), Institute of Semiconductor Technology (IHT), Technische Universität Braunschweig, Hans-Sommer Str. 66, 38106 Braunschweig, Germany; daniel.prades@tu-braunschweig.de

**Keywords:** gas sensor, NO_2_, micro-lightplate, graphene, TiO_2_

## Abstract

Low-power gas sensors that can be used in IoT (Internet of Things) systems, consumer devices, and point-of-care devices will enable new applications in environmental monitoring and health protection. We fabricated a monolithic chemiresistive gas sensor by integrating a micro-lightplate with a 2D sensing material composed of single-layer graphene and monolayer-thick TiO_2_. Applying ultraviolet (380 nm) light with quantum energy above the TiO_2_ bandgap effectively enhanced the sensor responses. Low (<1 μW optical) power operation of the device was demonstrated by measuring NO_2_ gas at low concentrations, which is typical in air quality monitoring, with an estimated limit of detection < 0.1 ppb. The gas response amplitudes remained nearly constant over the studied light intensity range (1–150 mW/cm^2^) owing to the balance between the photoinduced adsorption and desorption processes of the gas molecules. The rates of both processes followed an approximately square-root dependence on light intensity, plausibly because the electron–hole recombination of photoinduced charge carriers is the primary rate-limiting factor. These results pave the way for integrating 2D materials with micro-LED arrays as a feasible path to advanced electronic noses.

## 1. Introduction

Air quality assurance and environmental protection require monitoring of toxic and polluting gases with different spatial resolutions, from satellite surveillance to ground IoT networks and personal gas detection, which provide the most localized sensing [1,2]. Microsensors embedded in consumer devices, including mobile phones and wearables, would enable personal compliance with the cleanliness of the environment and, if exhaled air is detected, monitoring of the health situation [3]. A small footprint and low power consumption are of paramount importance for wearables and self-powered IoT devices. In this regard, chemiresistor-type gas sensors stand out for their potential for miniaturization and mass production in the semiconductor industry. Although the lowest power consumption can be achieved with sensors capable of reversible operation at room temperature [4], such devices frequently have very slow signal recovery and may not function stably enough in outdoor conditions without a miniature heater or light source.

Metal oxide-based sensors on MEMS platforms with micro-hotplates have reached a footprint of a few mm^2^ and power consumption of 10 mW [5,6]. The energy consumption is due to the heating required to accelerate the desorption of gases and bring the response and recovery times of the sensor within acceptable limits. An alternative to providing external energy for the amplification and acceleration of sensor responses is to use light instead of heat [7,8]. Light, especially if its quantum energy surpasses the bandgap energy of the sensor material, effectively produces electronic excitations that facilitate surface reactions.

Recently, it was demonstrated that instead of using a separate light source, an effective way to couple the light energy into the sensing material is to integrate it with a micro-LED (μLED) or, by analogy with a micro-hotplate, with a micro-lightplate (μLP) [9]. With ZnO nanoparticles coated on the isolating layer on top of a blue (455 nm) μLED’s active area (190 μm × 250 μm), NO_2_ gas was detected at 25 ppb with power consumption as low as 30 μW [10]. With a different design of a monolithic GaN-based μLED and sensing material composed of In_2_O_3_ nanowires, the power level was further reduced below the microwatt level, albeit at the expense of higher (1 ppm) NO_2_ concentration [11]. In Ref. [12], ultraviolet (UV) μLP (390 nm) was used, and a significant increase in power efficiency was achieved by reducing the size of the μLED from 200 μm to 30 μm. A NO_2_ limit of detection (LoD) of 15 ppb was demonstrated at a power consumption of ~200 μW.

The major driving forces for μLED development are the lighting and display markets, with the trends being a reduction in power consumption and pixel size of the arrays for micro-displays [13]. Beyond display technology, the integration of LED nitride and CMOS technologies may open avenues for groundbreaking applications such as highly efficient nanosensors and miniaturized neuromorphic networks [14]. Chemical sensors and sensor arrays [15] may benefit from the miniaturization trend if the technology for forming sensor materials on top of the LED array keeps track. The thickness of the metal oxide (MOX) layers in the μLP devices described above was 100s nm, and it is typically even more in commercial hotplate-based solutions. For light-assisted sensors, the power can be relatively easily reduced by shrinking the area of the sensor. This, in turn, means that thinner sensing layers are needed to match the small μLED size (≤3 μm [16]) as well as to ensure efficient light absorption in the sensing layer.

An excellent opportunity for this could be the use of 2D materials [17,18], which are not only atomically thin and have a large surface-to-volume ratio, an essential prerequisite for the material’s high gas sensitivity, but can also be technologically handled for mass production [19]. Graphene is an excellent transducer of environmental perturbations because of its low density of charge carriers, and it has the high electrical conductivity needed for microscopic sensing areas [20]. Pristine graphene, however, has low gas sensitivity because of its inertness (e.g., the binding energy of NO_2_ is only approximately 0.06 eV [21]) and has to be functionalized by introducing defects, functional groups, or nanoparticles [22,23,24,25]. It was demonstrated that the sensitivity of graphene could be increased by two orders of magnitude by adding a nanolayer of ZrO_2_ via pulsed laser deposition [26]. In such a layered heterostructure, graphene acts as a transducer, owing to its low electron density but high conductivity, and the metal oxide serves as a receptor for gas molecules. Recently, an even more efficient heterostructure of single-layer graphene with TiO_2_, a well-known photocatalytic material [27], was shown to be perfectly suitable for air quality applications, owing to its optimal sensitivity and high stability [28]. An efficient charge transfer from graphene to NO_2_ molecules adsorbed on titania was demonstrated.

In this study, we developed a monolithic gas sensor by integrating a graphene-TiO_2_ heterostructure (Gr/TiO_2_) with UV μLP and demonstrated the detection of NO_2_ gas at typical concentrations for air quality monitoring (20–150 ppb) at low power levels (electrical power down to 100 μW, optical power < 1 μW). We analyzed the performance characteristics related to gas response power dependence and discussed the underlying processes.

## 2. Experimental Section

### 2.1. Device and Sensor Material Fabrication

**Figure 1:**
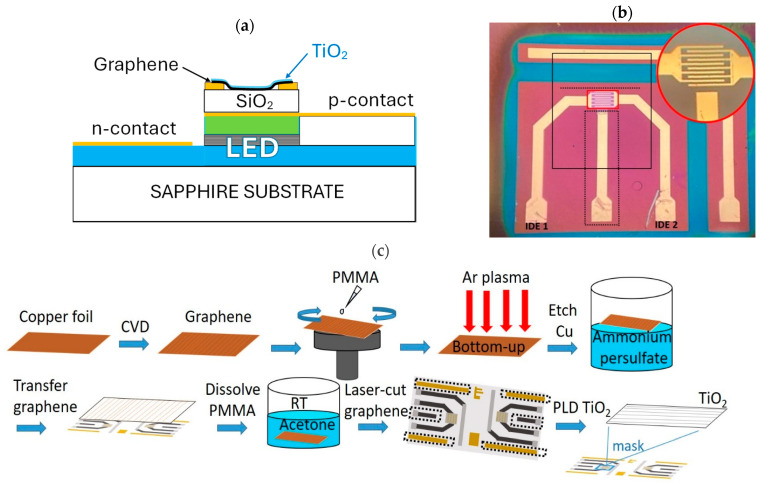
(**a**) Schematic cross-section of the device, (**b**) photograph of the μLP with a magnified area of interdigitated electrodes, and (**c**) sequence of sensor layer fabrication on the μLP.

The InGaN-based μLPs had a layout similar to that in Ref. [8] with a μLED and interdigitated electrode (IDE) area of 190 μm × 250 μm. A schematic cross-section and a photograph of the device are shown in Figure 1a,b, respectively. The μLPs grown on sapphire wafers were diced in pairs, and the dimensions of a die with two devices on it are 4.05 × 5.6 mm^2^. Single-layer CVD graphene grown on a polycrystalline copper foil (Graphenea, San Sebastian, Spain) was transferred onto the μLP die using a wet transfer procedure [Figure 1c]. After cutting the graphene/Cu/graphene sheet with an appropriate size and covering it with a layer of PMMA, argon plasma treatment (Diener Tetra 30/50, Plasma Surface Technology, Ebhausen, Germany) was applied to remove the graphene from the uncovered side of the sheet. The next step was to remove copper from the PMMA/graphene/Cu sheet by keeping it in a 1 M ammonium persulfate (Sigma-Aldrich, Steinheim, Germany) solution for two hours. The PMMA/graphene film was then rinsed several times with deionized water, transferred onto the μLP in water, and left to dry overnight. Finally, the μLP with the transferred PMMA/graphene layer was baked at 120 °C for an hour and then placed in pure acetone (≥99.8%, Thermo Fisher Scientific, Seelze, Germany) for 2 h to dissolve the polymer layer. After drying the device, a part of the graphene was selectively removed from its surface to galvanically separate the LED and sensor circuits (see Figure 1b). The electrical isolation between the sensor and LED circuits was accomplished in two stages: by a short 1 min Ar plasma etching while masking the middle area around the interdigitated electrodes (IDE) and then by laser cutting of graphene near the IDE. The black rectangle in Figure 1b shows the masked area during plasma etching for graphene removal, and the dotted lines show the laser-cutting trajectory. A femtosecond laser (ORIGAMI O-05LP, Istanbul, Turkiye) with a pulse energy density of 0.7 mJ/cm^2^ was used to cut a few μm wide path through graphene without damaging the underlying μLP. Thereafter, pulsed laser deposition (PLD) was applied to produce a functionalizing layer on top of the graphene. Before deposition, the μLPs with graphene were heated in a PLD chamber at 150 °C in a vacuum (10^−6^ mbar) for 1.5 h. PLD was performed at 45 °C in 0.05 mbar N_2_ gas using a KrF excimer laser (COMPexPro 205, Coherent Lambda Physic GmbH, Göttingen, Germany) with a pulse frequency of 5 Hz and an energy density of 5 J/cm^2^ for target ablation. The number of laser pulses used for target ablation was 100, which resulted in a TiO_2_ layer with a thickness of about 0.5 nm [28].

### 2.2. Gas Response Measurement Set-Up

**Figure 2:**
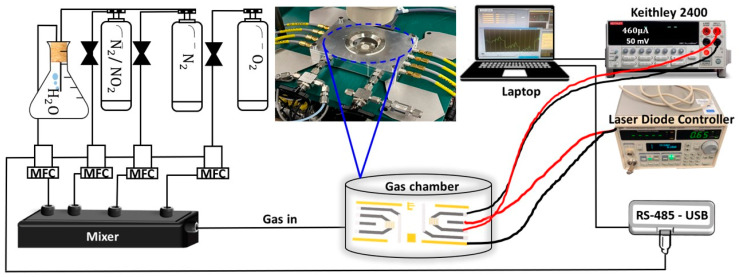
Schematic illustration of the gas sensing setup.

The experimental setup for the gas sensitivity measurements is shown in Figure 2. The test gas was prepared from cylinder gases (N_2_, O_2_, NO_2_/N_2_ of 99.999% purity, AS Linde Gas, Tallinn, Estonia), which passed through mass flow controllers (model SLA5820, Brooks Instrument, Hatfield, PA, USA) into a 180 cm^3^ micro-probe chamber (Nextron, Busan, Korea). The total gas flow rate through the chamber was maintained at 200 sccm. The O_2_ content in the gas mixture was kept constant at 21% to simulate a typical atmospheric composition. The relative humidity of the testing gas was held either at 0, 20, or 40% by bypassing part of the N_2_ through the water bubbler. In the cross-sensitivity tests, the gases were supplied from cylinders, except for ozone, which was produced with a UV lamp-based generator (SOG-1, UPV/Analytic Jena, Jena, Germany) and monitored using an analyzer (model 430, Teledyne API, San Diego, CA, USA). The electrical conductance of the sensors was measured with a Keithley 2400 source measure unit, using a typical 50 mV bias voltage.

### 2.3. Characterization Methods and Instruments

The sensor materials were characterized by scanning electron microscopy (Nova NanoSEM 450, FEI, Hillsboro, OR, USA) and Raman spectroscopy (inVia, Renishaw, Gloucestershire, UK; 514.5 nm excitation). The electrical current of the μLP was regulated with a laser diode controller LDC500 (SRS, Sunnyvale, CA, USA, and the optical power was measured using a model 1918-C power meter (Newport, Irvine, CA, USA). The μLP optical power was evaluated from the measured data by assuming that the source was Lambertian. The electroluminescence spectrum was recorded using FLAME-T-XR1-ES spectrometer (Ocean Optics, Largo, FL, USA; spectral resolution < 2 nm).

## 3. Results and Discussion

### 3.1. Characterization

**Figure 3:**
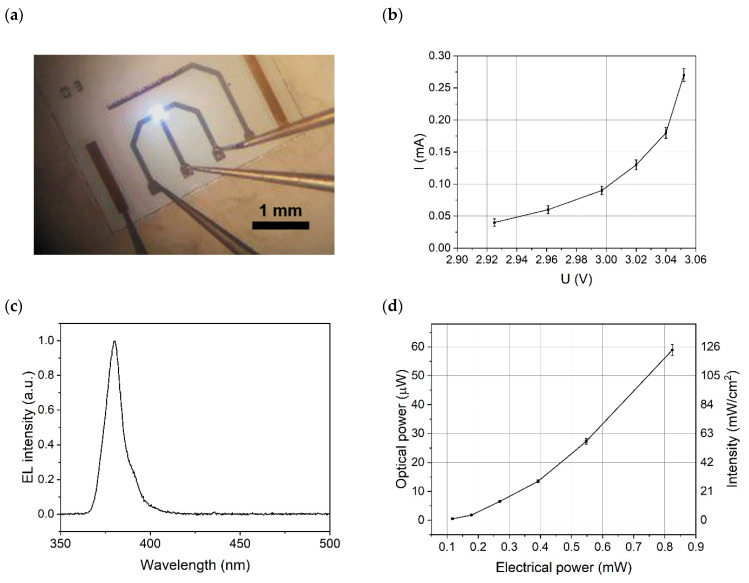
(**a**) Image of the μLP with a working LED, its above-threshold (**b**) volt–ampere characteristic, (**c**) electroluminescence (EL) spectrum, and (**d**) dependence of the μLP optical power and surface intensity on the applied electrical power.

The characteristics of the μLP are shown in Figure 3, where (a) shows the working device with microprobe contacts and panel (b) plots its volt–ampere characteristic curve. The peak emission wavelength of the μLP was 380 nm [see the spectrum in Figure 3c]. Figure 3d shows the optical power and intensity on the sensor area versus the consumed electrical power. The external quantum and wall-plug efficiencies had incidentally very similar numerical values, being 0.4 ± 0.04% near the threshold current of 40 μA and increasing to 7% at higher currents.

**Figure 4:**
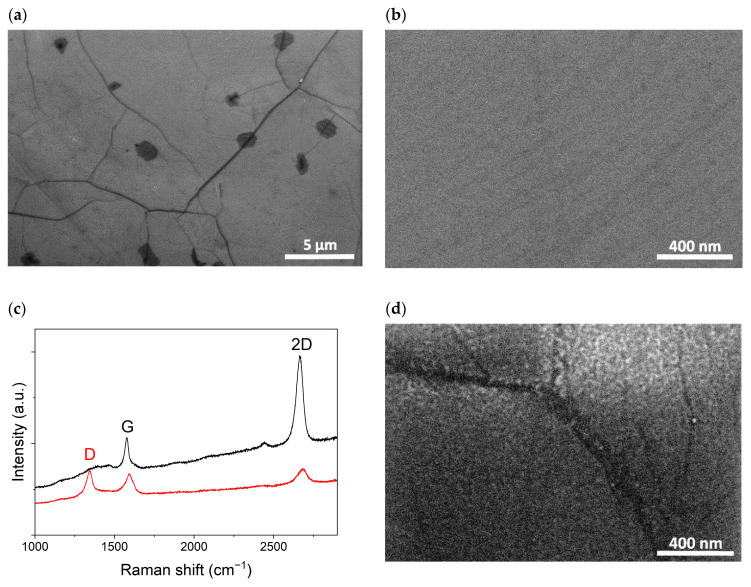
(**a**,**b**) SEM images of CVD graphene on μLP. (**c**) Raman spectra of graphene before and after the PLD of TiO_2_. (**d**) SEM image of the sensor material after coating the graphene with a TiO_2_ nanolayer.

SEM images of graphene in the electrode gap of the μLP are shown in Figure 4a,b. The spots that are darker in color in Figure 4a are commonly observed in CVD graphene and are related to the multilayer graphene. The dark linear features, which are also commonly observed, are due to the topography of the polycrystalline Cu-foil used in the synthesis process and the grain boundaries of graphene. The Raman spectrum [Figure 4c] contains mainly two bands, a G-band at 1577 cm^−1^ and a 2D-band at 2664 cm^−1^, with an intensity ratio of 1:3, which is characteristic of single-layer graphene [29]. The XPS spectra of the graphene used in the present study were recently analyzed in detail in Ref. [30]. Deconvolution of the C1s peak resolved sp2 carbon (87%), sp3 carbon (7.5%), and oxidized (C–O, C=O, O=C–O) species (5–6%). After the PLD of TiO_2_ on top of graphene, a defect-related band D (1344 cm^−1^) emerges in the Raman spectrum. The SEM image [Figure 4d] shows the granular morphology of the deposited material, uniformly coating the graphene. The linear features observed in the pristine graphene image “shine” through a thin TiO_2_ coating and are also visible in Figure 4d. For comparison, Figure 4b shows an image of pristine graphene at exactly the same scale, which does not have a granular oxide layer on top but instead shows characteristic streaks formed by the copper substrate during growth.

### 3.2. Gas Sensing Performance

**Figure 5:**
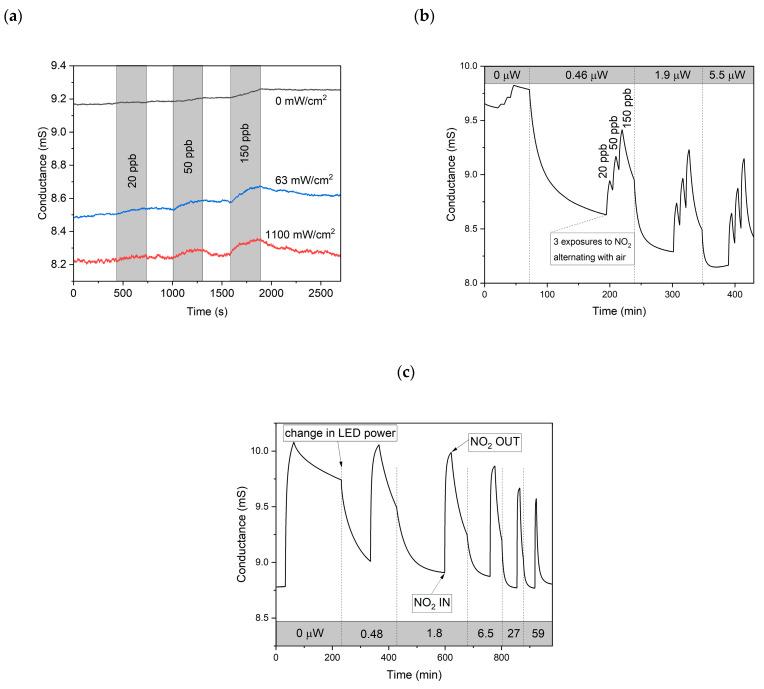
(**a**) Dynamic responses of pristine graphene to NO_2_ gas at concentrations of 20, 50, and 150 ppb at different irradiation intensities on μLP. (**b**) The same for the Gr/TiO_2_ μLP sensor, recorded in the dark and under incremental UV illumination with the μLED optical power of 0.46, 1.9, and 5.5 μW (corresponding to 0.8, 3.3, and 9.7 mW/cm^2^). Synthetic air was used as the background gas. (**c**) Sensor conductance during the exposures to 150 ppb of NO_2_ gas at different levels of μLP optical power. The power levels in μW units are shown in the gray area at the bottom. Synthetic air with a relative humidity (RH) of 20% was used as the background gas.

The sequences of conductance measurements when the sensor was exposed to a series of NO_2_ concentrations in the dark and under μLED illumination are presented in Figure 5a,b for pristine graphene and Gr/TiO_2_, respectively. The measured conductance is solely due to graphene in both cases, and the approximately monolayer-thick TiO_2_ (bandgap 3.2 eV [25]) plays a negligible role. NO_2_ gas was injected at three different concentrations (20, 50, and 150 ppb), each for 5 min, followed by 5 min intervals in clean air. The responses of the device with pristine graphene [Figure 5a] were relatively small (~1%) even under high intensity (~1 W/cm^2^) of UV light. The insensitivity of pristine (defect-free) graphene to toxic gases has been observed in several previous publications [22,28,31].

The sequence in Figure 5b starts with NO_2_ gas exposures in the dark; three relatively small raising steps in conductance can be seen. CVD graphene is p-doped by adsorbed oxygen and water molecules in air [32,33], and MOX-coated graphene retains this conductivity type [26]. Because NO_2_ acts as an electron acceptor during adsorption, hole doping is further promoted in the presence of NO_2_, increasing the conductivity. However, the gas response in the dark was slow and showed prolonged recovery. After switching on the μLED at its lowest power (see Figure 4b; 70 min after the start of the measurement), the conductance of the samples held in synthetic air starts to decrease and stabilizes at a lower level. Such a persistent photoresistance effect has also been observed in pristine graphene and is explained by the photoinduced removal of oxygen and the associated decrease in the density of electron holes in graphene [34]. It can be seen from the figure that under the influence of UV radiation, the gas responses become faster, and the recovery is improved with increasing optical power. The values of the applied optical power are indicated on top in Figure 5b as being directly relevant to the photoinduced phenomena; the values of the electrical power applied were 0.12, 0.18, and 0.24 mW, respectively.

To investigate the dependence of photoinduced processes on light intensity, measurements were made with the same NO_2_ concentration but at different irradiation powers [Figure 5c]. Longer clean air intervals between the gas injections were used for more complete signal recovery. This figure shows a relatively strong gas response under dark conditions, unlike that shown in Figure 5b. The difference is that the sensor was exposed to UV shortly (an hour) before the measurements, whereas in the case of Figure 5b, the sensor was held in the dark for several days. Persistent photoresistance can, indeed, last many hours [33] and seemingly also affects the surface properties of Gr/TiO_2_ with respect to NO_2_. The processes occurring in the sensor material can be summarized in simple terms as follows:

(1) $$\mathrm{Gr}/{\mathrm{TiO}}_{2}+h\nu \;\to \;e^{-}+h^{+}\;$$

(2) $$O_{2}^{-}(\mathrm{ads})+h^{+}\;\to \;O_{2}\left(\mathrm{gas}\right)$$

After the photoelectrons and holes are formed, the previously chemisorbed oxygen is partly released, and the hole conductivity decreases. As a result, more sites were becoming available for NO_2_ adsorption, written as follows:

(3) $$\;{\mathrm{NO}}_{2}(\mathrm{gas})+e^{-}\;\to \;{\mathrm{NO}}_{2}^{-}\left(\mathrm{ads}\right)$$

This process is more dominant than oxygen adsorption because of the significantly larger electron affinity of NO_2_ (2.273 eV) than that of O_2_ (0.450 eV) [35]. The recovery of conductance was due to the NO_2_ desorption process, written as follows:

(4) $$\;\;{\mathrm{NO}}_{2}^{-}(\mathrm{ads})+h^{+}\;\to \;{\mathrm{NO}}_{2}\left(\mathrm{gas}\right)$$

Clearly, both the response and recovery processes became faster with increasing light power, as seen in Figure 5.

For quantitative characterization, the response and recovery curves were approximated using biexponential kinetics, written as follows:

(5) $$\;\;\;\;\;\;\;\;\;\;\;\;\;\;\;\;\;\;\;\;\;\;F\left(t\right)=A_{0}+A_{1}\left(1-exp\left(-k_{1}t\right)\right)+A_{2}\left(1-exp\left(-k_{2}t\right)\right)$$

The average response and recovery rate *k_av_* was defined as follows:

(6) $$\;\;k_{av}=\frac{A_{1}k_{1}+A_{2}k_{2}}{A_{1}+A_{2}}$$

We used average parameters, allowing us to compare the data in cases where the fitting procedure converged to only a single exponent in Equation (5). Moreover, there is a distribution of rates on an amorphous metal oxide surface [36], rather than just one or two discrete values. The two kinetic components are still a reasonable approximation, although they are somewhat dependent on the time span of the approximated curve.

**Figure 6:**
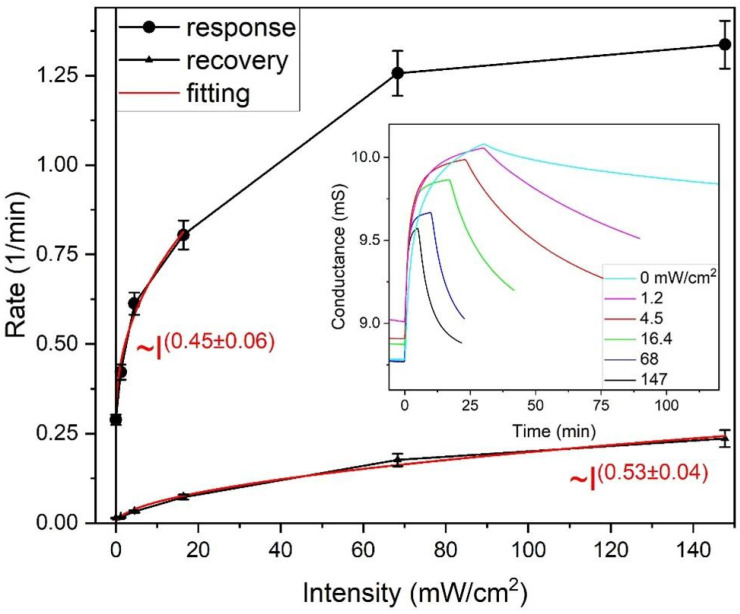
The dependence of average response and recovery rates on light intensity. Approximations with power functions and power exponents of the intensity (I) dependence are shown in red. The inset shows the response curves approximated with Equation (5).

Initially, the response rates to the step-like NO_2_ gas injection increase rapidly with the increasing light intensity (Figure 6). The rate dependence can be fitted with the power dependence, but it clearly reaches a plateau above 60 mW/cm^2^. Saturation can be considered an artifact because it is actually determined by the instrumental gas exchange rate, which can be estimated from the chamber volume and the flow rate to be slightly above 1 min^−1^. The recovery process was slower and could be fitted with a power law for the entire range of light intensities. Both approximations using the power law led to exponents close to 0.5.

This dependence can be explained as follows. Let us assume that the surface processes involving NO_2_ are described by the Langmuir kinetic model, written as follows:

(7) $$\frac{d\theta }{dt}=k_{a}\left(1-\theta \right)-k_{d}\theta$$

where *k_a_* and *k_d_* are adsorption and desorption rates, respectively, and *θ* is the coverage of occupied adsorption sites. The processes (3) and (4) imply the following:

(8) $$k_{a}=a\cdot n_{e}\cdot p$$

(9) $$k_{d}=d\cdot n_{h}$$

where *n_e_* and *n_h_* are the density of electrons and holes, respectively, and *p* is the gas pressure. The parameters *a* and *d* have constant values at a given temperature.

The general solution of Equation (7) is written as follows:

(10) $$\;\theta \left(t\right)=\theta \left(0\right)\cdot e^{-kt}+\frac{k_{a}}{k}\cdot \left(1-e^{-kt}\right)$$

where

(11) $$\;\;k=k_{a}+k_{d}=a\cdot n_{e}\cdot p+d\cdot n_{h}$$

and *θ* (0) is the initial coverage at *t* = 0.

If, in addition, we assume that only photogenerated charge carriers participate in adsorption–desorption processes and the carriers are annihilated by the bimolecular process [37] much faster than the adsorption–desorption processes, then we obtain the following:

(12) $$\;\;\frac{dn_{e}}{dt}=\frac{dn_{h}}{dt}=I\kappa -bn_{e}n_{h}$$

where *I* is photon flux, *κ* is the absorption constant, and *b* is the annihilation constant. As the equilibrium in Equation (12) is established much faster than in Equation (7), we can assume the following:

(13) $$0=\frac{dn_{e}}{dt}=\frac{dn_{h}}{dt}=I\kappa -bn_{e}n_{h}$$

and evaluate the quasi-static values of charge carrier densities for Equations (7)–(11):

(14) $$n_{e}=n_{h}=\sqrt{\frac{I\kappa }{b}}$$

Consequently, the rates *k_a_*, *k_d_*, and *k* in Equations (8), (9) and (11) are proportional to the square root of the light intensity. This result persists for a more general case of inhomogeneous Langmuir adsorption, for example for two (or more) adsorption sites, and consequently, the average rates (Equation (6)) behave similarly in accordance with the experimental results shown in Figure 6.

The total response amplitude *A*_1_ + *A*_2_ obtained by fitting decreased by approximately 25% in the full light intensity range spanning from 1 to 150 mW/cm^2^, mainly at the expense of the slower component. This differs from the behavior of MOX-based sensors coated directly on the μLP, which showed a bell-shaped (log-normal) dependence of the response amplitude on light intensity [10,12]. Obviously, an optimal intensity exists in the latter case corresponding to the bell-curve maximum. For the sensor studied in this work, there seems to be no optimal intensity owing to the physics of the device; the optimum intensity is determined by practical considerations (a trade-off between the response and recovery rates vs. power consumption).

Another difference between graphene-based materials and semiconducting metal oxides is the significantly higher conductivity of the former. As a result, the electrodes can be accommodated in a much smaller area, and measurements can be made with a higher signal-to-noise ratio. In our measurements, the sensitivity was S = 25 μS/ppb at the lowest studied concentration, whereas the rms noise amplitude (N) was typically only 0.2 μS within a 1 Hz bandwidth. If estimating the level of detection (LoD) of NO_2_ with a common rule LoD = 3N/S (≥99% confidence level), the result is LoD = 0.024 ppb.

**Figure 7:**
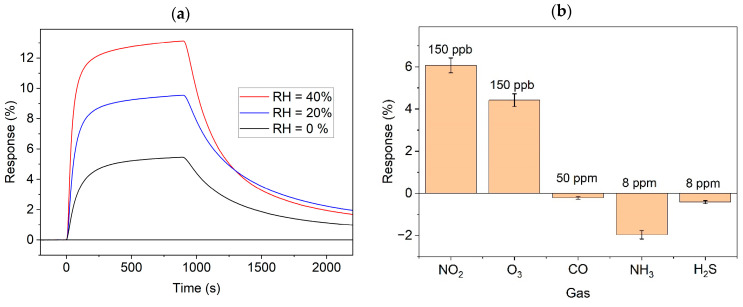
Relative responses to (**a**) 150 ppb of NO_2_ at different levels of relative humidity and (**b**) different toxic gases at concentrations as indicated.

Finally, to characterize the selectivity, we checked the cross-sensitivity to several other toxic gases and humidity (Figure 7). Panel (a) compares the dynamic relative responses (relative change in conductance) to NO_2_ gas at three different RH levels at the irradiation intensity of 60 mW/cm^2^. The responses appeared to be somewhat stronger and faster in the humid air. The impact of humidity is a common and complex phenomenon in chemiresistive sensors [38]. In a recent study [39], the dependence of the light-assisted response to NO_2_ on humidity was ascribed to the decrease in active sites and increase in carrier concentration owing to adsorbed water molecules and OH groups. The latter factor also explains our results through the deactivation of the recombination centers by adsorbed water molecules.

The relative conductance changes during 15 min of gas exposure to different toxic gases in dry air under 5 mW/cm^2^ irradiations are shown in Figure 7b. As expected, the sensitivity to O_3_ was similar to that of NO_2_, as both molecules are strong oxidizers, with electron affinities of 2.103 and 2.273 eV, respectively [34]. In the case of the reducing toxic gases CO, NH_3_, and H_2_S, the concentrations had to be several orders of magnitude higher in order to observe sizeable effects.

## 4. Conclusions

In summary, we assembled monolithic gas microsensors by integrating a UV micro-lightplate with a 2D sensing material made of CVD graphene and a less than a nanometer thick layer of TiO_2_. The low-power (0.5 μW optical, 100 μW electrical) operation of the device was demonstrated while detecting NO_2_ concentrations typical of air quality monitoring with an extrapolated limit of detection of 0.02 ppb. The gas response amplitude was nearly constant over the studied light intensity range (1–150 mW/cm^2^) because of the balance between the photoinduced adsorption and desorption processes. The rates of both the response and the recovery processes followed an approximately square-root dependence on the light intensity, implying that bimolecular electron–hole recombination is the primary mechanism of the photoinduced charge carrier relaxation and the rate-limiting factor of the sensor. In practical terms, such a dependence on light intensity is useful because there is relatively little loss in reaction speed when reducing power. The integration of 2D materials with high-density μLED arrays can provide a feasible path for advanced electronic noses with large sensor arrays.

## Data Availability

Data will be made available upon request.
